# The experience of device failure after cochlear implantation

**DOI:** 10.1186/s40463-023-00652-7

**Published:** 2023-07-17

**Authors:** Jeong Heon Kim, Yeonjoo Choi, Woo Seok Kang, Hong Ju Park, Joong Ho Ahn, Jong Woo Chung

**Affiliations:** Department of Otorhinolaryngology-Head and Neck Surgery, Asan Medical Center, University of Ulsan College of Medicine, Seoul, South Korea

**Keywords:** Cochlear implantation, Device failure, Prodromic symptoms, Reimplantation, Audiologic performance

## Abstract

**Background:**

The present study describes the treatment of patients at a tertiary institution who experienced device failure after Cochlear Implantation (CI), as well as identifying prodromic symptoms that could assist in the timely identification and management of device failure.

**Study design:**

Retrospective database review (January 2000–May 2017).

**Setting:**

Single tertiary hospital.

**Methods:**

Factors recorded included the etiology of hearing loss; age at first and revision CI surgeries; surgical information, including operation time and approach; electrical outcomes after implantation; device implanted; symptoms of device failure; history of head trauma; and audiologic outcomes as determined by categories of auditory performance (CAP).

**Results:**

From January 2000 to May 2017, 1431 CIs were performed, with 27 (1.9%) undergoing revision surgeries due to device failure. The most common etiology of hearing loss was idiopathic (12/27), followed by cochlear hypoplasia (5/27). Mean age at initial CI was 11.8 (1–72) years, with 21 being pre-lingual and 6 being post-lingual. Of the total devices initially implanted, 80.5% were from Cochlear, 15.9% from MED-EL, and 3.5% from Advanced Bionics. The failure rates of these devices were 1.3%, 3.1%, and 10.0%, respectively. The most suggestive symptom of device failure was intermittent loss of signal. Mean CAP scores were 5.17 before reimplantation and 5.54 and 5.81 at 1- and 3-years, respectively, after reimplantation.

**Conclusion:**

The most suggestive symptom preceding device failure was intermittent loss of signal. Patients who present with this symptom should undergo electrical examination for suspected device failure. Audiologic outcomes showed continuous development despite revision surgeries.

## Background

Cochlear implantation (CI) is regarded as an effective method of audiologic rehabilitation for patients with moderate to severe sensorineural hearing loss (SNHL) who achieved little to no benefit from conventional hearing aids (HA). Since its initial introduction in 1961, CI has been widely used for audiologic rehabilitation worldwide [1, 2]. Improvements in surgical techniques and implant devices have enabled more patients with SNHL to regain their hearings through CI. Although these devices are designed to last a lifetime, some patients experience implant failure [3, 4].

Overall explantation rates have been reported to range from 4 to 10% [5–7]. The reason of explantation may be categorized into hard failure, soft failure, and other medical reasons. Hard failure refers to a measurable device failure leading to malfunction, whereas soft failure indicates a decline in implant function without any demonstrable fault in the device [8]. Other medical reasons refer to operative site problems, such as wound infection, device migration, and extrusion of the electrode array or receiver [9]. Rates of revision surgery due to device failure have been reported to be relatively rare. Batuk et al. reported a total of 127 revision CI out of 2181 cases (5.8%), while Battmer et al. reported 173 reimplants out of 3417 cases of CI (5.1%). These previous studies have explored the potential outcome of device failure of CI, but have mostly focused on the rate of failure itself and the trend of failure according to age [10, 11]. Since device failure is a rare event, we endeavored to include audiologic outcomes and prodromic symptoms, based on the 18 years of experience at our institution, to evaluate the value of a timely revision surgery in terms of audiologic outcomes.

The progression of audiologic outcome following revision surgery after device failure in CI is unclear. Several studies have reported better audiologic outcome after revision surgery [12–16], whereas one found that speech perception scores declined after reimplantation [17].

The present study describes the treatment of a large number of patients who underwent CI at a tertiary medical center, as well as reporting the rate and treatment of those who experienced device failure. This study was also designed to identify any prodromic symptoms of device failure that could facilitate early detection, minimizing the time gap during which patients experience hearing impairment, particularly avoiding the disruption of language development in pre-lingual patients.

## Material and methods

### Patients and methods

The medical records of 1431 patients who were diagnosed with moderate to severe SNHL and underwent CI from January 2000 to May 2017 at our tertiary institution were retrospectively reviewed. Surgery was performed by 6 different surgeons in our facility. Patients who received initial cochlear implantation during the period above were included in this study regardless of age, sex, onset of hearing loss, and underlying anomalies. Patients who received implantation with devices from manufacturers other than Cochlear, MED-EL, and Advanced Bionics were excluded due to their scarcity. Furthermore, explantation cases due to reasons other than device failure were not included in our study group. Other reasons include surgical site infection and cerebrospinal fluid leakage. The mean age at primary cochlear implantation was 19.1 (1–80) years, 912 (63.7%) patients were under the age of 18 (interquartile range; 2–8), and 519 (36.3%) patients were adults (interquartile range; 31–56). Patients who underwent reimplantation after initial CI due to device failure were identified. Patient profiles and intraoperative and post-operative parameters were recorded, including the etiology of hearing loss, ages at first and revision CI surgeries, and surgical information including operation time and approach. Also recorded were the type of device and their electrically evoked compound action potentials (ECAPs), including neural response telemetry (NRT) for Cochlear devices, auditory nerve response telemetry (ART) for MED-EL devices, and neural response imaging (NRI) for Advanced Bionics devices. Other factors recorded included symptoms of device failure, history of head trauma, and audiologic outcomes, such as categories of auditory performance (CAP) scores and speech discrimination.

This study protocol was approved by the Institutional Review Board of Asan Medical Center (study protocol 2021-1415), which waived the requirement for informed consent due to the retrospective nature of this study.

### Device assessment

Device failure was evaluated according to European consensus guidelines. Hard and soft failures were defined in accordance with the Cochlear Implant Soft Failures Consensus Development Conference Statement [8].

Devices from three manufacturers were implanted into patients at our institution from 2000 to 2017. Of the 1431 devices implanted, 1152 (80.5%) were from Cochlear, 227 (15.9%) were from MED-EL, and 52 (3.6%) were from Advanced Bionics. Since 2019, we have implanted 3 devices manufactured by Oticon, and have not been reported of any device failure related to this brand so far.

### Performance assessment

Patients who underwent CI were audiologically evaluated during follow-up, with patients requiring reimplantation undergoing audiologic evaluation before revision surgery and 1 and 3 years after reimplantation. Audiologic evaluation included word and sentence recognition tests, CAP, and speech discrimination (SD). Because most of the enrolled patients underwent surgery at a pre-lingual age, audiological performance was initially assessed by measuring CAP score, which was the main pre- and post-operative assessment. CAP scores consist of eight categories, with 0 indicating no awareness of environmental sounds, 1 indicating awareness of environmental sounds, 2 indicating response to speech sounds, 3 indicating identification of environmental sounds, 4 indicating discrimination of speech sounds, 5 indicating understanding of common phrases without lip-reading, 6 indicating understanding of conversations without no lip-reading, and 7 indicating ability to use a telephone with a known speaker [18].

To further evaluate audiologic performance, patients were administered open set word tests, which assessed the percentage of correctly identified one- and two-syllable words. Adult post-lingual patients (n = 6) were also evaluated using SD tests.

### Device inspection

All devices removed from patients who underwent revision surgery were returned to their original manufacturer for evaluation. Reports were obtained for 25 of the 27 removed devices.

### Statistical analysis

Device survival was determined using the Kaplan–Meier method and compared by log-rank tests. CAP scores were compared using Wilcoxon signed rank tests and CAP scores were correlated with age using Spearman correlation analysis. All statistical analyses were performed using the Statistical Package for Social Sciences 23 (SPSS Inc., Chicago, IL, USA), with *p*-values < 0.05 considered statistically significant.

## Results

From January 2000 to May 2017, 1431 CIs were performed at our tertiary institution, with 27 cases (1.9%) being confirmed with device failure (26 patients; one patient received bilateral revision). All cases diagnosed with device failure went through pre-operative CT scans, which did not reveal any particular abnormality in all cases and proceeded to explantation and revision CI. Table 1 shows the demographic characteristics of these patients. Mean age at initial implantation was 11.8 (1–72) years; of the 27 cases, 21 were pre-lingual and six were post-lingual. Their most common otologic anomalies were idiopathic (44.4%), cochlear hypoplasia (18.5%), bony cochlear nerve canal narrowing (14.8%), enlarged vestibular aqueduct syndrome (14.8%), Mondini dysplasia (14.8%), and incomplete partition type 1 (7.4%). The average time from initial CI to confirmed device failure was 35.8 ± 32.6 months, and the mean time from recognition of device failure to revision CI was 33.5 ± 26.7 days. Altogether, the mean time interval from initial to revision CI was 37.0 ± 33.0 months.

**Table 1:** Demographic and clinical characteristics of included patients

Characteristics	No. (N = 27)
Age (year) at initial CI	11.8 (1–72)
Adults (%)	5 (18.5)
Children (%)	22 (81.5)
Age (year) at revision CI	14.8 (2–72)
Sex (%)	
Male	17 (63.0)
Female	10 (37.0)
Side (%)	
Right	16 (59.3)
Left	11 (40.7)
Accompanying anomaly (overlap, %)	
Idiopathic	12 (44.4%)
Cochlear hypoplasia	5 (18.5%)
BCNC^a^ narrowing	4 (14.8%)
EVAS^b^	4 (14.8%)
Mondini dysplasia	4 (14.8%)
IP^c^ type 1	2 (7.4)
Language status (%)	
Pre-lingual	21 (77.8)
Post-lingual	6 (22.2)
Prodromic symptoms (within 3 months, %)	
None	13 (48.1)
Intermittency of sound	10 (37.0)
Abnormal impedance	4 (14.8%)
History of head trauma	2 (7.4)

^a^Bony cochlear nerve canal

^b^Enlarged vestibular aqueduct syndrome

^c^Incomplete partition

Fourteen (51.9%) cases had prodromic symptoms or signs within 3 months prior to device failure, whereas 13 (48.1%) did not. Of the 14 cases with prodromic symptoms, 10 experienced intermittency of sound, and four showed abrupt changes in impedance (increased or undetectable impedance). The features of each subject are specified in Table 2.

**Table 2:** Features of patients who experienced CI device failure

No	Manu-facturer	Model	Sex	Age (y)		Side	Language status	Anomaly	Prodromic symptom	Duration to (mo)		Head trauma	CAP		
				Initial	Revision					Failure	Revision		Before revision	1Y	3Y
1	Cochlear	CI24RE	M	2	3	R	Pre-lingual	Mondini, EVAS	–	9	10	No	5	7	7
2		CI422	M	9	15	L	Pre-lingual	EVAS	–	69	73	No	7	7	7
3		CI512	M	38	39	R	Post-lingual	CN hypoplasia	Intermittency of sound	10	11	No	7	7	7
4		CI512	M	8	9	L	Pre-lingual	–	Intermittency of sound	11	12	No	7	7	7
5		CI512	M	6	8	L	Pre-lingual	Mondini, EVAS	–	30	31	No	7	6	6
6		CI512	F	32	34	R	Post-lingual	–	–	30	32	No	7	7	7
7		CI512	F	13	15	L	Post-lingual	–	Intermittency of sound	31	33	No	7	5	7
8		CI512	F	4	5	L	Pre-lingual	–	Intermittency of sound	8	9	No	6	6	6
9		CI512	M	24	25	R	Pre-lingual	CN hypoplasia	Intermittency of sound	14	14	No	5	5	5
10		CI512	M	2	6	R	Pre-lingual	–	Intermittency of sound	50	51	No	6	6	6
11		CI512	F	6	8	R	Pre-lingual	CN hypoplasia, BCNC	Intermittency of sound	35	36	No	4	4	5
12		CI512	M	13	14	R	Post-lingual	–	Intermittency of sound	11	12	No	4	4	4
13		CI512	M	63	68	R	Post-lingual	–	–	63	63	No	–	–	–
14		CI512	M	3	7	R	Pre-lingual	Mondini	Intermittency of sound	48	49	No	0	5	6
15		CI512	F	4	5	L	Pre-lingual	–	Intermittency of sound	12	13	No	5	7	7
16	MED-EL	FLEX24	M	1	3	L	Pre-lingual	IP type I	Elevated impedance	18	20	No	4	4	5
17		FLEX24	M	1	4	R	Pre-lingual	IP type I	Undetectable impedance	31	32	No	4	5	5
18		FLEX28	F	2	3	R	Pre-lingual	BCNC	Undetectable impedance	8	9	Yes	2	5	5
19		FLEX28	F	1	2	L	Pre-lingual	CN hypoplasia, BCNC	Unstable impedence	13	14	No	4	5	6
20		FLEX28	M	73	73	R	Post-lingual	–	–	1	3	No	7	7	7
21		FLEX28	F	3	3	L	Pre-lingual	CN hypoplasia, Mondini, EVAS	–	1	2	No	1	6	7
22		FLEX28	F	2	5	L	Pre-lingual	BCNC	–	31	35	No	4	2	2
23	Advanced Bionics	EBP	M	3	12	R	Pre-lingual	–	–	113	113	No	7	7	7
24		HF.1.2	M	2	12	R	Pre-lingual	–	–	122	125	No	7	7	7
25		HiRes90K	M	2	5	R	Pre-lingual	EVAS	–	40	40	Yes	6	7	7
26		HiRes90K	M	2	8	L	Pre-lingual	–	–	73	74	No	5	5	5
27		HiRes90K	F	1	8	R	Pre-lingual	Mondini	–	82	85	No	2	2	2

Operative factors are summarized in Table 3. Mean durations of the initial and revision operations were identical (2.83 ± 0.04 vs. 2.95 ± 0.03 h, *p* = 0.883). In 23 (85.1%) cases, initial surgery consisted of device implantation via a cochleostomy approach, with all cases achieving full insertion of electrodes. During revision surgery, the round window approach in one case was converted to cochleostomy due to new bone formation and granulation tissue at the insertion site, whereas the cochleostomy approach in one case was converted to the round window approach. Electrodes were fully inserted in all revision cases. Readings of impedance and ECAP were normal in 92.6% and 63.0%, respectively, of initial cases, declining to 81.5% and 44.4%, respectively, during revision. The specific data for each patient are illustrated in Table 4.

**Table 3:** Operative findings of included patients

	No. (N = 27)	
	Initial CI	Revision CI
Time from initial to revision CI (mo)	37.0 ± 33.0	
Time from initial CI to recognition^a^ (mo)	35.8 ± 32.6	
Time from recognition to revision CI (day)	33.5 ± 26.7	
Operation time (hr)	2.83 ± 0.04	2.95 ± 0.03
Approach of CI (%)		
Round window (RW)	4 (14.8)	4 (14.8)
Cochleostomy	23 (85.2)	23 (85.2)
Electrode loading		
Full insertion	27 (100.0)	27 (100.0)
Partial insertion	0 (0.0)	0 (0.0)
Impedance^b^		
Full	25 (92.6)	22 (81.5)
Partial	2 (7.4)	5 (18.5)
ECAP (%)^c^		
Full	17 (63.0)	12 (44.4)
Partial	5 (18.5)	10 (37.0)
None	5 (18.5)	5 (18.5)
Surgical findings at revision		
Fibrous tissue		6 (22.2%)
Granulation tissue		4 (14.8%)

^a^ate of follow-up presenting with a confirmed device failure

^b^“Full” Impedance indicates normal measurements of impedance in all electrodes; “Partial” indicates abnormal measurement of impedance in at least one electrode

^c^“Full” ECAP indicates measureable thresholds in all electrodes; “Partial” indicates an unmeasurable threshold in at least one electrode

**Table 4:** Operative findings of patients who experienced CI device failure

No	Initial CI				Revision CI			
	Approach	Electrode loading	Impedance^a^	ECAP^b^	Approach	Electrode loading	Impedance	ECAP
1	Cochleostomy	Full	22/22	22/22	Cochleostomy	Full	22/22	22/22
2	Cochleostomy	Full	22/22	22/22	Cochleostomy	Full	22/22	22/22
3	Cochleostomy	Full	22/22	22/22	Cochleostomy	Full	22/22	22/22
4	Cochleostomy	Full	22/22	22/22	Cochleostomy	Full	22/22	22/22
5	Cochleostomy	Full	22/22	22/22	Cochleostomy	Full	22/22	1/22^c^
6	Cochleostomy	Full	22/22	22/22	Cochleostomy	Full	22/22	21/22^c^
7	Cochleostomy	Full	22/22	22/22	Cochleostomy	Full	21/22^c^	19/22^c^
8	Cochleostomy	Full	22/22	22/22	Cochleostomy	Full	22/22	22/22
9	Cochleostomy	Full	22/22	22/22	Cochleostomy	Full	22/22	22/22
10	Cochleostomy	Full	22/22	22/22	RW	Full	22/22	22/22
11	Cochleostomy	Full	22/22	0/22^c^	Cochleostomy	Full	22/22	0/22^c^
12	Cochleostomy	Full	22/22	22/22	Cochleostomy	Full	22/22	22/22
13	Cochleostomy	Full	22/22	0/22^c^	Cochleostomy	Full	22/22	0/22^c^
14	Cochleostomy	Full	22/22	22/22	Cochleostomy	Full	22/22	22/22
15	Cochleostomy	Full	22/22	19/22^c^	Cochleostomy	Full	22/22	22/22
16	Cochleostomy	Full	22/22	2/24^c^	Cochleostomy	Full	24/24	21/24^c^
17	Cochleostomy	Full	22/22	0/24^c^	Cochleostomy	Full	12/12	0/12^c^
18	RW^d^	Full	12/12	0/12^c^	RW	Full	12/12	5/12^c^
19	RW	Full	12/12	0/12^c^	Cochleostomy	Full	12/12	0/12^c^
20	RW	Full	12/12	5/12^c^	RW	Full	12/12	8/12^c^
21	RW	Full	9/12^c^	3/12^c^	RW	Full	12/12	10/12^c^
22	Cochleostomy	Full	12/12	12/12	Cochleostomy	Full	12/12	0/12^c^
23	Cochleostomy	Full	16/16	16/16	Cochleostomy	Full	16/22^c^	22/22
24	Cochleostomy	Full	16/16	16/16	Cochleostomy	Full	16/16	16/16
25	Cochleostomy	Full	12/16^c^	16/16	Cochleostomy	Full	13/16^c^	3/16^c^
26	Cochleostomy	Full	16/16	16/16	Cochleostomy	Full	15/16^c^	12/16^c^
27	Cochleostomy	Full	16/16	6/16^c^	Cochleostomy	Full	16/16	5/16^c^

^a^Impedance findings illustrated as No. of normal measurement electrodes out of total No. of electrodes

^b^ECAP findings illustrated as No. of electrodes with measureable threshold out of total No. of electrodes

^c^Partial impedance reading, non to partial ECAP reading highlighted

^d^RW; Round Window approach

**Fig. 1:**
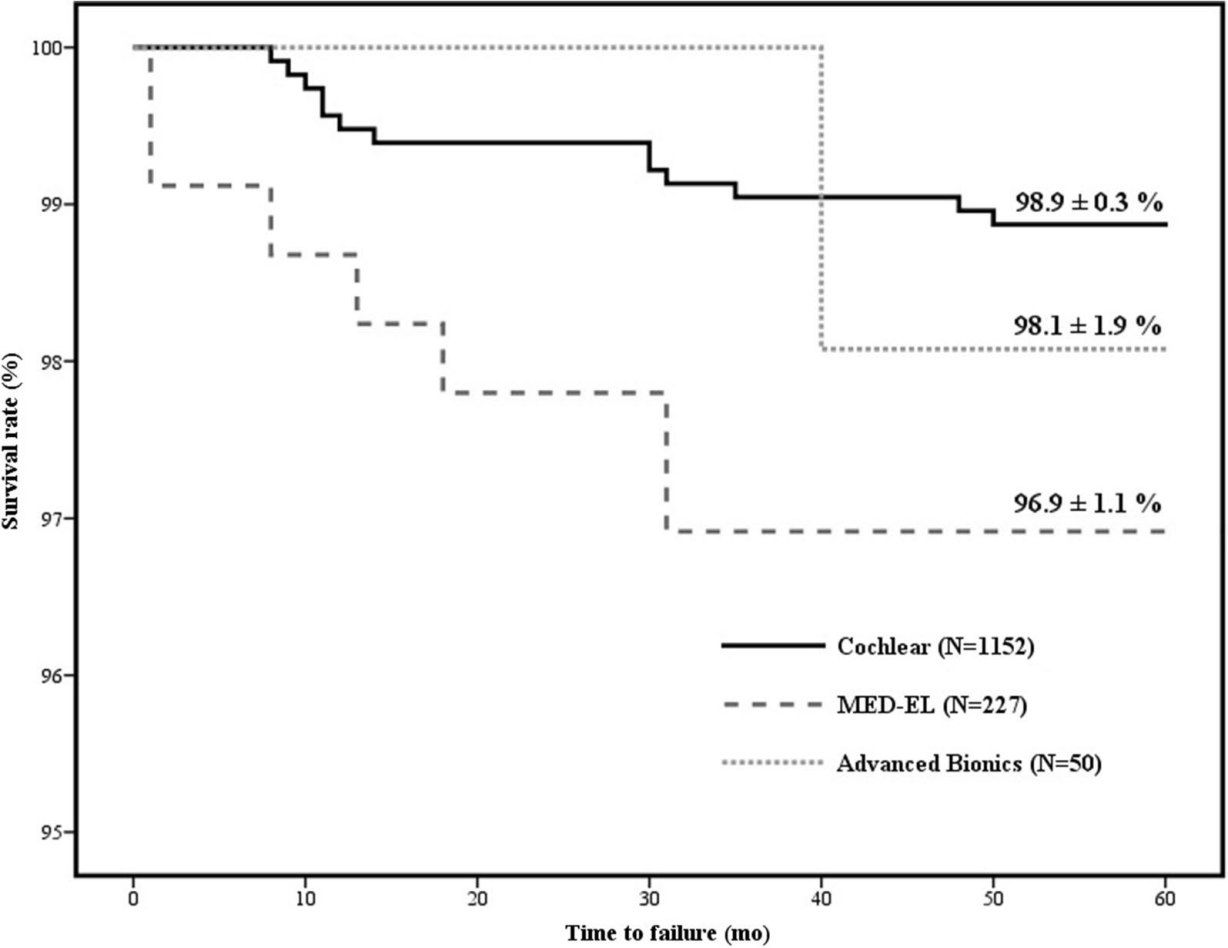
Kaplan–Meier analysis of 5-year overall device survival by device manufacturers

Of the devices initially implanted, 80.5% were from Cochlear, 15.9% were from MED-EL, and 3.6% were from Advanced Bionics. The overall failure rates of these devices were 1.3%, 3.1%, and 10.0%, respectively. Kaplan–Meier analyses showed that the 5-year survival rates of these three CI devices were 98.9 ± 0.3%, 96.9 ± 1.1%, 98.1 ± 1.9%, respectively, with the difference between survival rates of Cochlear and MED-EL devices being statistically significant (*p* = 0.023) (Fig. 1). With each failure taken into consideration, in order to achieve statistical power of 0.8, the minimal number of total cases of Cochlear and MED-EL are 2267 and 454.

**Fig. 2:**
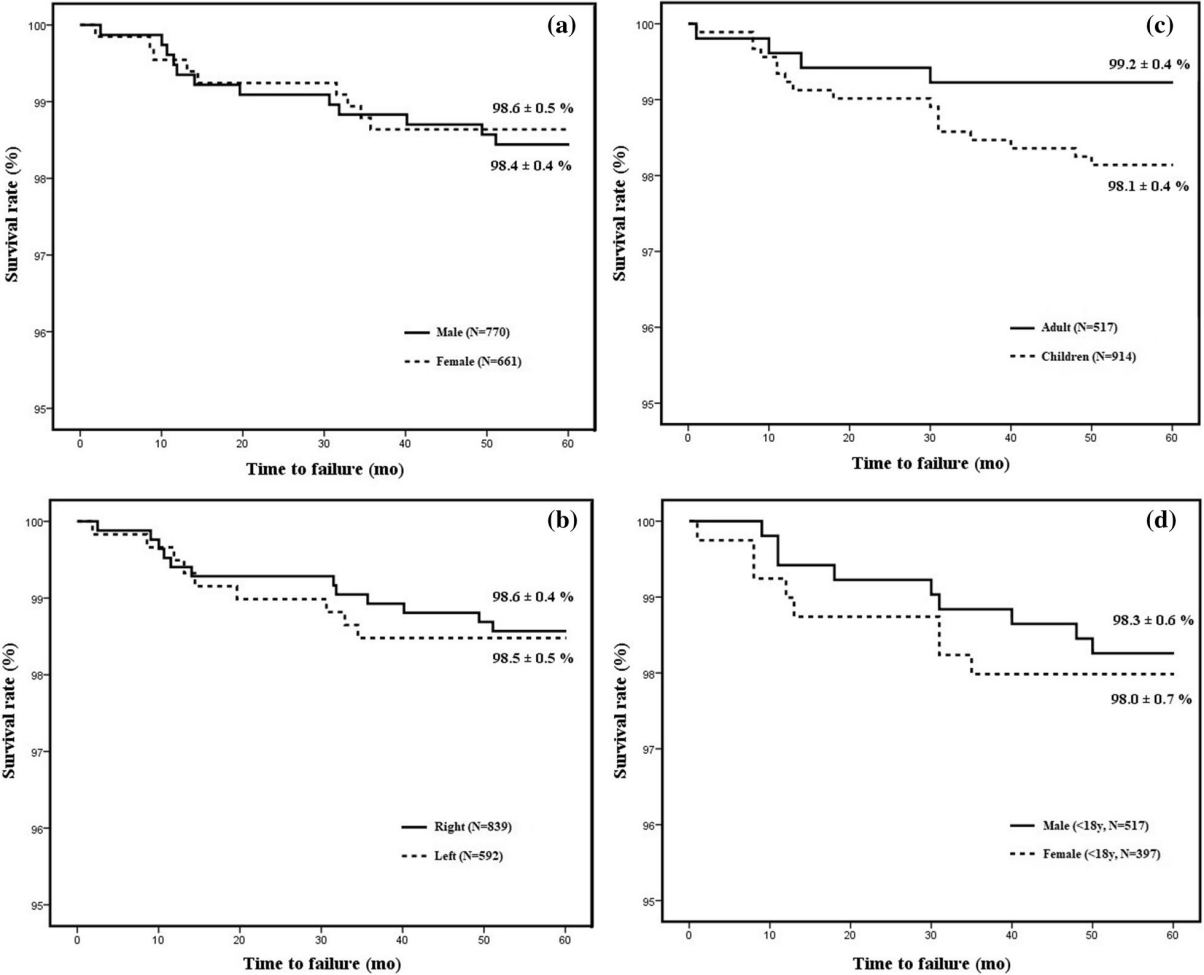
**a**. Kaplan–Meier analysis of 5-year overall device survival by sex. **b**. Kaplan–Meier analysis of 5-year overall device survival by side of operation. **c**. Kaplan–Meier analysis of 5-year overall device survival by age. **d**. Kaplan–Meier analysis of 5-year overall device survival by sex within the pediatric group

We have further differentiated our study group depending on various factors and compared the 5-year device survival according to each variable; sex, side of operation, type of electrode, and age. Our study group was composed of 770 male and 661 female cases, with 5-year device survivals being 98.4% and 98.6% respectively (*p*-value 0.789) (Fig. 2a). Right side was more dominant in our study group (right 839, left 592), and 5-year device survivals were 98.6% and 98.5% (*p*-value 0.577) (Fig. 2b). When dividing into two major types of electrodes (perimodiolar versus straight) regardless of manufacturer, 963 were implanted with perimodiolar electrodes and 468 were implanted with straight electrodes. Five-year survivals were both 98.5% (*p*-value 0.726). When dividing our subjects according to age, 517 were adult cases while 914 were pediatric cases (under the age of 18 at time of implantation). Adult cases had a slightly higher 5-year survival rate than pediatric cases (99.2% and 98.1%) but did not have any statistical significance (Fig. 2c). We have also divided pediatric cases into male and female (517 male, 397 female) but also did not exhibit any statistical difference in 5-year survivals (98.3%, 98.0%, *p*-value 0.696) (Fig. 2d).

Beginning in March 2011, the N5 (CI 512) model was implanted into a total of 114 CI patients. The overall failure rate of the N5 devices was 11.4% (13 of 114), whereas the failure rate of all other devices was 1.1% (14 of 1317). The 5-year survival rates of the N5 series and all other devices were 89.5 ± 2.9% and 99.3 ± 0.2%, respectively (*p* < 0.001).

All 27 removed devices were returned to their manufacturers for evaluation. Reports were received for 25 of these devices, with 21 reports finding no demonstrable defects in these devices. Malfunctions were observed in the other four devices, with two showing malfunctions in the receiver-stimulator, and two having broken electrodes. Of the two devices with broken electrodes, one was removed from a patient with a recent history of head trauma, with the report on this device stating that the malfunction was likely due to this trauma.

**Fig. 3:**
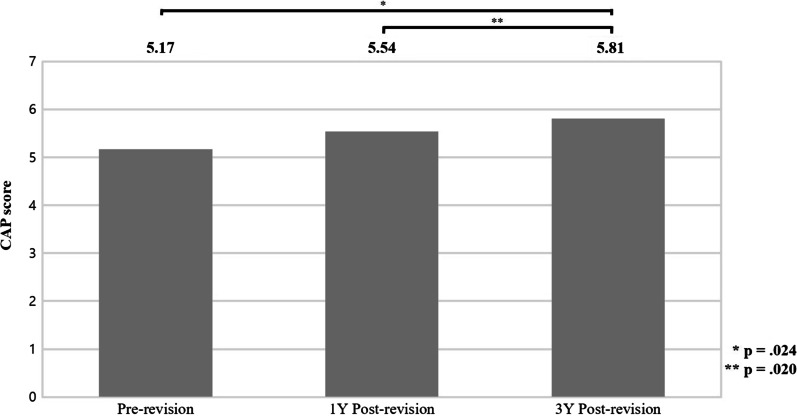
CAP scores before and 1 and 3 years after device implantation

CAP scores were measured before and 1 and 3 years after reimplantation, with all three measurements obtained for 26 of the 27 cases. Mean CAP scores at these times were 5.17, 5.54, 5.81, respectively, with Wilcoxon signed rank tests showing statistically significant differences between pre-implantation and 3-year post-implantation scores (*p* = 0.024) and between 1- and 3-year post-implantation scores (*p* = 0.020), suggesting a continuous improvement in audiologic function over time (Fig. 3).

Open set word tests were performed before and 1 and 3 years after reimplantation, with all three measurements obtained for 11 of the 27 cases. Results of one- and two-syllable tests were analyzed separately using Wilcoxon signed rank tests. On one syllable word tests, pre-implantation scores did not differ significantly from 1-year (*p* = 0.932) and 3-year (*p* = 0.325) post-implantation scores, nor were there differences between 1- and 3-year post-implantation scores (*p* = 0.151). Although pre-implantation scores on two-syllable word tests did not differ significantly from 1-year (*p* = 0.462) and 3-year (*p* = 0.726), the 1- and 3-year post-implantation scores differed significantly (*p* = 0.041), indicating a slight improvement over time after revision surgery.

Six post-lingual patients were evaluated by SD tests before and 1 and 3 years after revision surgery. Only two of these patients displayed steady improvement in SD over time, with one having scores of 16%, 28%, and 40%, respectively, and the other having scores of 36%, 48%, and 48%, respectively. The other four patients had invariable results over time, with one having scores at these time periods of 52%, 48%, and 52%, one having constant scores of 36%, and two having constant scores of 0%.

Differences in CAP scores between periods were analyzed to uncover any relationship between age and hearing improvement. The gaps between pre-implantation and 1-year post-reimplantation scores, pre-implantation and 3-year post-implantation scores, and 1- and 3-year post-reimplantation scores were each analyzed with Spearman’s correlation test. The differences between pre-implantation scores and 1-year (correlation coefficient − 0.41, *p* = 0.007) and 3-year (correlation coefficient − 0.48, *p* = 0.002) post-implantation scores showed significant negative linear correlations with age.

## Discussion

This study is based on 18 years of experience with CI device failure at our tertiary hospital. The overall device failure rate was 1.9%. Ulanovski et al., Wang et al. and Lane et al. reported a device failure rate of 2.0%, 4.8%, and 1.6%, respectively, showing similar results with the present study [9, 19, 20]. The time gap between diagnosis of device failure and revision surgery was small (33.5 ± 26.7 days), a crucial factor for language development, especially in pre-lingual patients [21].

Increases in the number of CIs in patients with SNHL are accompanied by increased numbers of explantation and reimplantation [22]. Device failure, whether hard or soft, is difficult to diagnose. Although the Cochlear Implant Soft Failures Consensus Development Conference Statement has defined soft failure, malfunctions cannot be detected without thorough evaluation, which is impractical to perform on a routine basis [23]. Several studies have therefore attempted to identify symptoms of device failure [9, 24–27].

Several clinical manifestations are suggestive of device failure. These include audiologic symptoms, such as atypical tinnitus, buzzing, and popping sounds; non-auditory symptoms, such as pain at the implant site and facial stimulation; performance issues, such as a sudden drop or slower decrement in hearing over time or intermittent performance; and mapping problems, such as changes in impedance, loss of channels, and changes in pulse width or duration [3]. The present study revealed similar findings, such as a suddenly non-functioning device, intermittency of function, and abnormal ECAP results. These findings reinforce the importance of identifiable symptoms and signs as indications of device failure and the need for early revision surgery.

Of the 27 cases with device failure, two, aged 2 and 7 years, reported a history of head trauma shortly prior to being diagnosed with device failure. Neither of these children, however, sustained damage to the bony skull or brain after the head trauma. Trauma may lead to device failure [28, 29], with the tendency of young children to fall and get injured, leaving them vulnerable to a higher risk of trauma-associated device failure [12, 13, 28, 30]. Most of the patients in the current study with device failure were children (21 children, 5 adults). Thus, despite the small proportion of patients with a history of head trauma, these findings suggest an inclination to device failure in children, which is in need of further investigation in future studies.

Patients who experienced explantation due to reasons other than device failure were excluded from this study. There have been a handful of cases where patients had no choice but to remove their devices because of other surgical problems such as infection or cerebrospinal fluid leakage. A total of 5 patients went through explantation due to such surgical complications, 4 with surgical site infection, and one with cerebrospinal fluid otorrhea. Although surgical complications also account for an important aspect in cochlear implantation, these cases were excluded since they do not correspond with our aim in the present study.

Revision surgery was complicated by the presence of granulation and fibrous tissue during the removal of defective implants, with altered anatomy during revision surgery frequently encountered [15]. Of the 27 cases of revision, 6 (22.2%) had fibrous tissue and 4 (14.8%) had granulation tissue attached to surrounding bone in the inlet area of the electrode. Fibrous tissue may result from the fibrous obliteration of the cochleostomy or round window area in the previous surgery. In revision surgery, fibrous tissue covered the insertion site after removal of the electrode. The fibrous and granulation tissue were successfully removed and the electrodes fully inserted in all 27 revision cases.

Device failure rate differs among manufacturers. The overall reimplantation rates of the Cochlear, MED-EL, and Advanced Bionics devices were reported to be 6.1%, 1.1%, and 8.2%, respectively, with their 10-year cumulative survival rates being 99.2%, 99.4%, and 93.9%, respectively (*p* < 0.0001) [20]. Although the Advanced Bionics device showed the highest revision rate (6.2%) and the MED-EL device had the highest 5-year cumulative survival rate, these rates did not differ significantly from rates observed following implantation of devices from other manufacturers, including Cochlear and Oticon [7]. Although the order of survival rates in the present study did not precisely match those in previous studies, the Advanced Bionics had the highest overall failure rate (9.8%). However, most Advanced Bionics devices were implanted between 2000 and 2010 (43 cases), with all the explanted devices from this manufacturer implanted during the same period, suggesting a possible alternative trend. The 5-year cumulative survival rates also differed among manufacturers, with survival rates differing significantly following implantation of Cochlear and MED-EL devices. However, due to our small patient group and the infrequent nature of device failure, the significant discrepancy between the survival of Cochlear and MED-EL is statistically underpowered as the number of cases do not meet the required minimum in order to satisfy the power of 0.8 (required number of Cochlear and MED-EL; 2267 and 454). Therefore, a larger group of cases will be needed in future studies to accurately evaluate the survivals by manufacturers.

We have also analyzed 5-year survivals depending on several demographic factors; sex, side of operation, type of electrode, and age. However, no specific variable seemed to have a significant impact on survival, with a minor exception of age (adult versus pediatric under 18 year of age). Although statistically insignificant (*p*-value 0.215), adult cases graphically seemed to have a better 5-year survival rate compared to pediatric cases. More cases of failure may be required in order to yield a more prominent discrepancy, but this may attribute to the tendency of children being more frequently exposed to undetectable trauma.

Several previous studies have assessed the implantation of the N5 (CI512) model. Shortly after being introduced into the market in 2010, this device was found to possess tightness issues, causing water leakage into the electronic components and leading to its recall in 2011 [6, 7]. The CI512 model was reported to account for 28% of hard failure cases, whereas another study found that the CI512 model had a revision rate of 19.7%, the highest among all devices tested [6, 7]. Our present results were similar, with the CI 512 having a failure rate of 11.4%. A comparison of 5-year cumulative survival between the N5 model and all other models showed that the N5 model had a significantly lower survival rate than the others. The mean duration of failure in the 13 cases of CI 512 device failure was 27.3 ± 18.0 months, with 5 (38.5%) devices failing within 1 year after surgery. These results support the need to closely monitor patients who have been implanted with the N5 model, with those showing declining function promptly undergoing revision surgery.

Because many of the patients in the present study were pre-lingual children, it was challenging to accurately evaluate their audiologic functions after revision CI. Several tests have been designed to evaluate audiologic function in patients, including monosyllabic and bisyllabic word tests, sentence tests, and CAP scores [8, 9]. Because of the limited amount of data, CAP score was the primary parameter for audiologic evaluation.

Comparisons of CAP scores before and after reimplantation showed statistically significant increases, particularly between pre-implantation and 3-year post-reimplantation scores and between 1-year and 3-year post-reimplantation scores. These results indicate that, despite the temporary gap of communication development at the time of device failure, overall audiologic progression was not meaningfully disrupted by discontinuation of hearing function.

The present study also evaluated the correlation between CAP score improvement and age. Differences between pre-implantation and 1-year post-reimplantation CAP scores and between pre-implantation and 3-year post-reimplantation CAP scores showed significant negative correlations with age; that is, younger patients tended to have a larger improvement in CAP score. This finding emphasizes the importance of revision CI, especially for younger patients, to enhance audiologic outcomes.

The results of open set word tests and SD tests were similar. Both tests revealed little to no significant differences from before to 1- and 3-years after revision surgery. These findings indicate that revision CI operation did not have any negative impact on patients diagnosed with device failure.

These findings are in agreement with the results of previous studies, which found that patients who underwent revision CI showed constant improvement in speech recognition scores. Marlowe et al. assessed pediatric patients who received re-implantation with an 18-month interval using the open-set instrument and demonstrated an increase in performance in 87% of patients. Eskander et al. employed the Pediatric Ranked Order Speech Perception (PROSPER) score composed of parental questionnaire, closed-set test, and open-set test. They illustrated that audiologic performance either improved or remained stable in 62% of patients. Gosepath et al. assessed patients with narrow-band noise audiometry (frequencies between 250 and 4 kHz), and threshold improved by 2.4 dB in general. Henson et al. however, reported that patients with better original sentence speech recognition results turned out to show a likelihood of decrease in sentence speech recognition score [13, 15–17]. Compared to previous studies, the present study somewhat lacks sufficient data on perioperative audiologic performance, as our main assessment is with CAP score. However, this study still holds an advantage in that not only did we compare pre- and post-operative audiologic performance on a statistical basis but have further revealed that these findings are correlated with patient’s age.

This study has a number of limitations such as the retrospective nature of this study, and our rather underpowered statistics due to the rarity of the event of cochlear implant device failure. The bimodal age distribution may also limit the application of our results to a general population. Furthermore, because most of the enrolled patients were pre-lingual children, CAP score was the only option for audiologic evaluation. Although CAP scores differed significantly from before to after revision surgery, the inclusion of the results of other tests, such as word and sentence tests, would have provided a more accurate output. Also, device failure in one ear may not affect audiological performance in bilateral CI users because the contralateral ear alone may be responsible for audiological development [31]. Despite the listed limitations, our study still has value in that it is composed of a large group of patients with sufficient follow-up period. We have also introduced possible prodromic symptoms which could assist in prompt identification of device failure, and have showed that a timely revision is linked to a better audiologic outcome especially in younger patients.

## Conclusions

Of the 1431 cases of CI over 18 years, 27 (1.9%) experienced device failure. The most common prodromic symptom was intermittent loss of signal. Timely assessment and management of these issues requires the identification of relevant symptoms that may precede device failure. Appropriate revision CI can result in the continuous development of overall audiologic functions.

## Data Availability

The datasets used and analysed during the current study are available from the corresponding author on reasonable request.

## Abbreviations

CI: Cochlear implantation
SNHL: Sensorineural hearing loss
HA: Hearing aids
ECAPs: Evoked compound action potentials
NRT: Neural response telemetry
ART: Auditory nerve response telemetry
NRI: Neural response imaging
CAP: Categories of auditory performance
SD: Speech discrimination
SPSS: Statistical package for social sciences
PROSPER: Pediatric ranked order speech perception
